# Experimental Investigation into the Mechanical and Piezoresistive Sensing Properties of Recycled Carbon-Fiber-Reinforced Polymer Composites for Self-Sensing Applications

**DOI:** 10.3390/polym16172491

**Published:** 2024-08-31

**Authors:** Bum-Jun Kim, Il-Woo Nam

**Affiliations:** 1 Spatial Design and Engineering, Handong Global University, Pohang-si 37554, Republic of Korea; skyrunner3217@handong.ac.kr; 2 School of Spatial Environment System Engineering, Handong Global University, Pohang-si 37554, Republic of Korea

**Keywords:** recycled carbon fiber, composite material, mechanical properties, sensing properties, fiber-reinforced composite material

## Abstract

This study investigates the mechanical and piezoresistive sensing properties of recycled carbon-fiber-reinforced polymer composites (rCFRPs) for self-sensing applications, which were prepared from recycled carbon fibers (rCFs) with fiber lengths of 6, 12, 18, and 24 mm using a vacuum infusion method. Mechanical properties of the rCFRPs were examined using uniaxial tensile tests, while sensing characteristics were examined by monitoring the in situ electrical resistance under cyclic and low fatigue loads. Longer fibers (24 mm) showed the superior tensile strength (92.6 MPa) and modulus (8.4 GPa), with improvements of 962.1% and 1061.1%, respectively. Shorter fibers (6 mm) demonstrated enhanced sensing capabilities with the highest sensitivity under low fatigue testing (1000 cycles at 10 MPa), showing an average maximum electrical resistance change rate of 0.7315% and a gauge factor of 4.5876. All the composites displayed a stable electrical response under cyclic and low fatigue loadings. These results provide insights into optimizing rCF incorporation, balancing structural integrity with self-sensing capabilities and contributing to the development of sustainable multifunctional materials.

## 1. Introduction

Carbon-fiber-reinforced plastic (CFRP) is popular in various fields for its lightweight and superior mechanical properties [1,2,3]. This composite material exhibits high strength, rigidity, and stiffness, as well as excellent resistance to temperature, corrosion, fatigue, and impact [4,5,6,7,8,9].

Compared to other fiber-reinforced composites, such as glass-fiber-reinforced plastic (GFRP) [10,11,12,13,14] and basalt-fiber-reinforced plastic (BFRP) [15,16,17], as well as metals and steels [18], CFRP exhibits superior characteristics, including tensile and flexural properties, resistance to corrosion [10,13,18] and fatigue [19], and specific strengths, making it a suitable substitute for traditional industry materials [4,20,21,22,23,24,25,26,27,28,29,30].

Furthermore, its high strength-to-weight ratio renders it an ideal material for practical use in high-tech industries such as aerospace, military, automotive, wind energy, construction, and medical and sports industries [1,2,3,4,7,9,20,21,22,23,24,25,26,27,31,32,33,34,35,36,37,38]. For high-performance composites used in critical applications, epoxy resins are used as a matrix in CFRPs due to their excellent adhesive properties, mechanical strength, and resistance to environmental degradation compared to other thermosetting resins, like polyester or vinyl ester, and thermoplastic resins, such as polypropylene or polyamide [39].

The market for carbon fibers (CFs) and related products, such as CFRPs, is growing significantly owing to the global demand from various high-tech industries for lightweight and high-performance materials [4,6,9,31,33,34,35,38,40,41,42,43,44]. However, this growth has the disadvantage of increasing CFRP waste [4,6,9,41,44,45,46,47,48,49,50,51]. Although, at present, landfills are the primary method for disposing of CFRP waste [4,7,9,50,52,53], this method has a negative environmental impact. Waste resin generates toxic products and pollutes the soil and water [4,6,31,43,54]. The estimated increase in the volume of CFRP waste presents a significant challenge for the future. To address this issue, governments worldwide have banned landfill disposal of CFRP waste [4,31,43,54]. Political decisions have led to increased attention from industries, governments, and academia to the development of recycling technologies for CFRP waste [3,36,37,40,43,44,52,55,56,57,58,59,60,61,62,63].

Recycling CFRPs is challenging because of their complex structure and chemical bonds [64]. Nevertheless, researchers have been developing recycling methods to reduce their environmental impact, reduce costs, and create new products. These methods include pyrolysis, solvolysis, mechanical grinding, and microwave heating. By recycling CFRPs, resources can be conserved, waste can be reduced, and value-added products can be created.

The high production costs and challenges of recycling have hindered the widespread adoption of CFRPs, despite their high performance potential [7,20,32,65]. Recycled CFRPs (rCFRPs) offer several advantages over conventional CFRPs, including lower costs and reduced environmental impact [7,53]. Compared to the production of CFs from raw materials, the production of rCFs from waste CFRPs is cost-effective and reduces the amount of CF waste [4,41,64,66,67,68]. The use of rCFs can significantly lower the manufacturing costs of CFRPs [7,20,69] while maintaining most of their mechanical properties [7,38,64]. According to the review by Manan and Nam on the application of rCFs to composites, the cost of rCFs is 15% of the production cost of virgin carbon fiber, making rCFs an attractive, economical reinforcement material for composites in high-tech industries [68]. Meng’s team estimates the cost of rCF to be below USD 5 per kilogram [67]. Thus, a rCFRP is a superior alternative to CFRP for various applications requiring lightweight and robust mechanical properties.

CFRP is electrically conductive, which also makes it suitable for structural health monitoring and damage detection. Electrical conductivity is a fundamental property of CFRPs that affects their behavior in diverse situations, including lightning strikes, electromagnetic interference, and self-sensing. Self-sensing is the ability of a material to detect strain or damage by measuring changes in its electrical resistance. The sensing ability of CFRPs can help monitor structural health and detect damage to structures [70].

Researchers have investigated the tensile properties and electrical sensing characteristics of the CFRPs for use in sensors [70]. They have also explored the mechanical properties of rCFRPs. However, research on the electrical properties of rCFRPs is insufficient. This represents a significant gap in our understanding of rCFRPs and their potential application as sensors, which can be filled by investigating the mechanical and electrical sensing properties of rCFRPs. The obtained results can evaluate the hypothesis that rCFRPs can exhibit piezoresistive behavior and provide reliable strain and damage measurements in their structures. To address this academic demand, the three experiments conducted in this study have examined the electrical and mechanical properties of rCFRPs with variations in fiber length. The influence of fiber length on the mechanical and sensing performances of rCFRPs was investigated in this study.

In this study, the mechanical and electrical properties of rCF-reinforced polymer composites as a function of fiber length were investigated. To explore this, chopped rCFs of four different lengths (6, 12, 18, and 24 mm) were incorporated into the epoxy resin (EP) during the fabrication procedures. Tensile tests assessed the mechanical properties as a function of the fiber length, whereas cyclic loading tests with simultaneous resistance measurements measured the sensing properties along the fiber length; length was the basis for comparative data analysis. Additionally, the initial electrical resistance, fractional changes in electrical resistance, and gauge factor were evaluated.

## 2. Experimental Procedure

### 2.1. Materials

The composites were fabricated by adding rCFs to EP. The EP and hardener mixtures comprised a polymer matrix. For the experiment, Kinetix R181 EP and Kinetix H141 hardener were used, which were procured from ATL Composites Ltd. (Molendinar, Australia). The EP–hardener (4:1) weight ratio recommendation by the manufacturer was followed to achieve the best curing results. Based on the fiber length (6, 12, 18, and 24 mm), four groups of rCFs were used as reinforcement fillers.

The rCFs of varying lengths were commercially sourced from Catack-H Co. Ltd. (Hwaseong-si, Republic of Korea). The fibers were precision-cut to specified lengths using the supplier’s proprietary cutting equipment, which is designed to ensure consistency in fiber length. Therefore, the manufacturer’s quality control processes were relied upon to ensure uniformity of fiber lengths within each batch. The rCFs are originated from the chemical recycling method, which was developed by Lee et al. in 2020, wherein the EP was broken down in a CFRP with an aqueous sodium hypochlorite solution [71,72,73].

While conventional thresholds for mechanical performance typically range from 1 mm to 10 mm, previous studies on recycled carbon fibers (rCFs) in cement composites have used lengths between 1 mm and 30 mm [68]. Therefore, fiber lengths from 6 mm to 24 mm were investigated to optimize rCF performance in the composite.

Technical datasheets for the rCF and EP used in this study were provided by Catack-H Co., Ltd. and ATL Composites Ltd. The physical properties of all materials were provided by the manufacturers and are listed in Table 1 and Table 2.

### 2.2. Sample Preparation

All rCFRP specimens were prepared using the vacuum-assisted resin infusion molding technique [74,75], which may also be referred to as vacuum-assisted resin transfer molding [76,77] or the Seemann composite resin infusion molding process [76,78,79], depending on the standardization adopted in the previous literature. In this technique, the pressure difference between the resin reservoir and the cavity drives the flow of resin, effectively pushing it into the cavity instead of sucking it in [79].

At the bottom, a glass module with dimensions of 600 mm × 600 mm was placed as the basis for all fabrication processes. A tapeable mold release film measuring 550 × 550 mm was attached. This eased the release of the specimen plate from the glass module during demolding.

**Figure 1:**
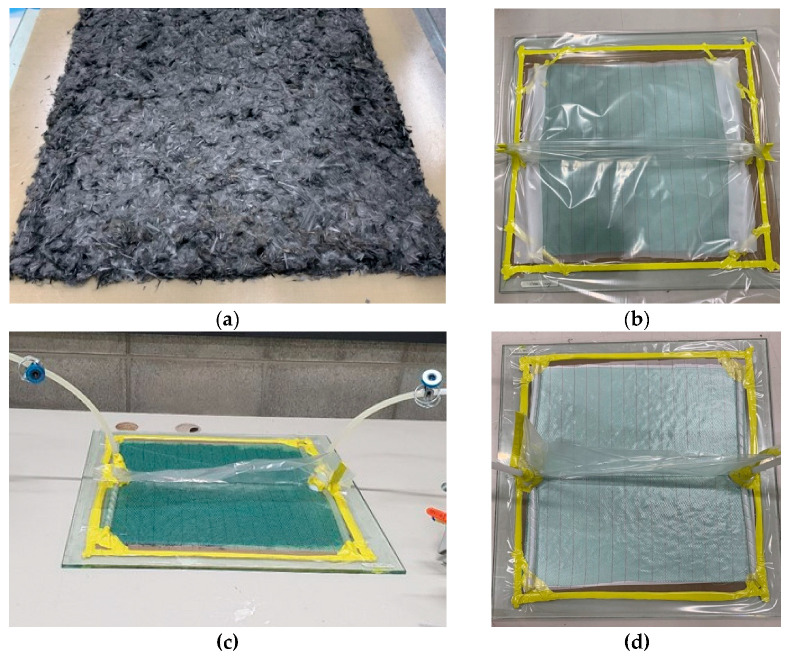
Images from the experimental process of (**a**) dry hand layout of rCF on the mold release film; (**b**) ready-to-be-vacuumed prefab covered with peel-ply, mesh, and vacuum bag; (**c**) EP-infused composite plate cured for 24 h at ambient temperature; and (**d**) vacuum status of the composite plate under a pressure of 1 bar.

A total of 80 g of rCF from a single rCF type among the four different fiber-length groups (6, 12, 18, and 24 mm) was weighed. The fibers were stacked in a square on a release film by hand. The rCF prefabs were randomly oriented (Figure 1). Further precaution was taken to prevent the rCF bundles from bunching during stacking by loosening them individually.

To ease the polymer demolding process, a thermoset polyester peel-ply (peel-ply; Aerofilm® PP230 Nylon 66 Peel Ply Red Tracer, Easy Composites Ltd, Stoke-on-Trent, United Kingdom) was cut into 500 mm × 500 mm pieces and laminated over an rCF prefab. A green polypropylene infusion mesh (mesh; Airtech Greenflow 75, Airtech Asia Ltd, Tianjin, China) with dimensions of 500 mm × 500 mm was then placed. This resin distribution medium facilitated the EP flow. Subsequently, a high-strength nylon release cloth of the same size was laminated to avoid air ingress and damage to the vacuum bag.

Along the surrounding boundary between the peel-ply and the glass molding plate, a sealant tape (Airtech AT-200Y, Airtech Asia Ltd, Tianjin, China) was tacked, which was used to secure the vacuum bags. Two spiral EP tube hoses (Spiral Tube SWP10, Kitagawa Industries Co., Ltd, Tokyo, Japan) with a length of 500 mm were attached with sealant tape, one at each end, to ensure the uniform distribution of EP.

The first step involved linking a tube connector (injection hub, infusion connector) to the center of the hose and attaching it to the sealant tape. The spiral hose was covered with a release cloth to protect the vacuum bag from being damaged. The vacuum bag was then placed over the release cloth and spiral hose and sealed with sealant tape to generate a vacuum inside. The sealant tape formed a T-shaped space on the stool of the specimen, where no spiral hose was present, to avoid further damage to the vacuum bag (Figure 1c). A small hole was made in the vacuum bag at the top of the injection hub to connect the injection and return the polyethylene tubing hoses, which were sealed with extruded tape.

The return hose was linked to the injection hub connected to the reservoir tank (tank), and another polyethylene tubing hose was connected to the tank using a rotary vacuum pump (pump). Both the return and injection hoses received clamps as attachments.

Shutting the clamps at the injection hose (input clamp) prevented air ingress, and the clamps at the reclaiming hose (output clamp) remained open. The bag then underwent a vacuum effect when the pump was initiated (Figure 1d). The pump was operated until the vacuum gauge in the tank indicated a pressure of 1 bar to remove the air inside the vacuum bag.

Closing the output clamps was followed by the pump shutdown, and the vacuum inside the bag was maintained for 15 min at 1 bar to confirm the vacuum preservation. The samples that met this criterion were EP-infused and subjected to specimen fabrication. The remaining samples were subjected to the same process again to ensure vacuum preservation.

After impregnation, the extra mixture was transferred to a resin trap through a vacuum hose. The EP infusion required reinitiation of the pump, and the injection hose was inserted into the EP container. The input clamp was then opened to infuse resin into the vacuum bag. After confirming that no air bubbles were present inside the vacuum bag with EP infusion, both clamps were shut, and the vacuum pump was switched off to preserve a tight seal. After 12 h of curing at ambient temperature, the rCF polymer composite plates were de-molded.

The rCFRP samples were made from plate-form composites, using compression molding or resin transfer molding. These processes create a quasi-in-plane random fiber orientation, where fibers predominantly lie in the plate’s plane with random distribution, and a vertical fiber orientation; through-plate thickness is minimal. Having the advantage of being able to preserve the original fiber orientation of the composites, water-jet cutting was used to obtain the specimens.

According to the ASTM D 3039 standard, the rCFRP plates were cut into rectangular strip plates with dimensions of 25 mm × 250 mm using an ultra-high-pressure water jet. While this technique may potentially introduce minor surface damage, it was chosen for its balance between preserving specimen integrity and practicality. Any potential effects from this cutting method are consistent across all sample groups, allowing for valid comparisons in this study [80,81,82,83,84,85,86,87,88,89].

### 2.3. Methodology

Three experiments were conducted to investigate the tensile and piezoresistive sensing properties of rCFRP with various rCF lengths. First, a longitudinal uniaxial tensile test using a universal testing machine (UTM) was performed to examine the tensile properties of rCFRPs. The fractured surface morphology of rCFRP specimens of post tensile test was examined using a field emission scanning electron microscope (FE-SEM, JSM-IT710HR, JEOL Ltd, Tokyo, Japan). Second, a cyclic loading test—which also utilized a UTM but with a digital multimeter (DMM) connected—was conducted to measure the electrical resistance. Third, a low fatigue test using the UTM with the DMM connected was performed to verify the continuous sensing characteristics.

All three experiments examined the mechanical and electrical sensing properties of the sensors at an ambient temperature. Each experiment involved the fabrication of a group of 16 specimens: four specimens of each of the four types of rCF were with lengths of 6, 12, 18, and 24 mm.

#### 2.3.1. Uniaxial Tensile Test

A UTM was used to perform the tensile test until specimen failure, according to the ASTM D3039 standard. The tensile strength was measured using displacement control at a crosshead speed of 5 mm/min. To evaluate numerous experimental specimens in a shortly given time, 5 mm/min suggested by the testing standard for a static test was followed for experiment conduction. ASTM D3039 states to define the loading rate so that the material fractures take 1 min to 10 min [90].

Ten thickness measurements of the specimen at ten equidistant points along the specimen were measured using a digital Vernier caliper. The specimens exhibited random thickness variations due to resin infusion, which is shown in Table 3. The average of these ten measurements was input into the control software of the UTM for the thickness value of each specimen, which was used for the stress calculation.

#### 2.3.2. Piezoresistive Sensing Performance Test

**Figure 2:**
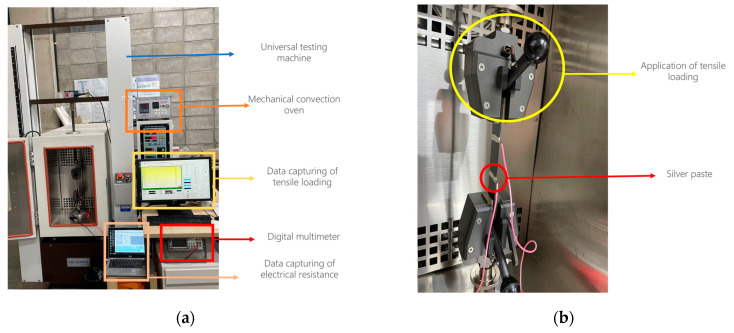
Test set-up for examining piezoresistive sensing: (**a**) digital multimeter was connected to an rCFRP specimen through conductive wires, and (**b**) the rCFRP specimen was mounted in UTM.

As Figure 2a visually represents, DMM was employed to monitor the variation in electrical resistance under cyclic loading. A strong and stable connection between the specimen and UTM is essential for obtaining reliable results. Instead of direct contact between the metal clamps, sandpaper was attached to both sides of the specimen.

The preparation for the electrical conductivity measurement involved applying the silver paste on a 60 mm interval and 5 mm spaced section at both ends of the specimen gauge length and wrapping it with copper wires. Figure 2b depicts the silver paste application and copper wire connections to the specimens. The insulating tape secured the copper wires and prevented gaps between the wires and the rCFRP specimen. The DMM measures the electrical resistance under a load via linked wires. DMM Viewer 2 software was used to save the data for analysis.

The specimens were securely tightened with the UTM grips to ensure maximum contact, thus minimizing triboelectricity caused by potential friction. By attaching an extensometer, the strain was measured at a target distance of 50 mm. The thickness of each specimen was measured six times using a digital Vernier caliper and then averaged. The measured thickness points were along the middle part of the sample with a width of 25 mm for a long cross-sectional length of 250 mm, and the section from 60 to 190 mm was divided into six equal parts. The tensile stress was measured using displacement control at a crosshead speed of 2 mm/min while piezoresistive test was conducted. Using UTM control software, five tensile cyclic loading experiments were designed with a maximum tensile stress of 10 MPa based on the average thickness of each sample. A copper wire and the DMM in the two-wire mode were attached to both specimen ends. The UTM and DMM simultaneously recorded the variation in electrical resistance with loading for five cycles, and the electrical resistance was monitored throughout the experiment.

After the test, the load and stress values were exported to Microsoft Excel and merged with a file that recorded the initial electrical resistance and its change. Using these merged files, the average values of the initial electrical resistance and maximum rate of change in the electrical resistance were computed and compared. The experiment yielded the change in electrical resistance according to the increased and decreased cyclic loads.

The experimental conditions for the low-fatigue tensile test were the same as those for the piezoresistive sensing performance test under five cyclic loading tests, except for the number of cycles where each specimen was subjected to 1000 cycles, meeting the minimum requirements for low-fatigue tensile tests.

## 3. Results and Discussion

### 3.1. Mechanical Properties

**Figure 3:**
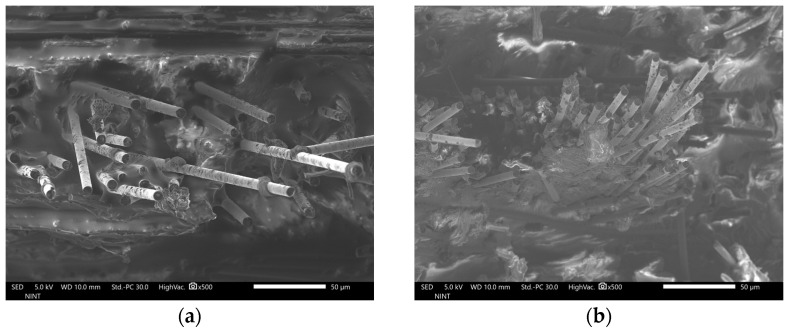

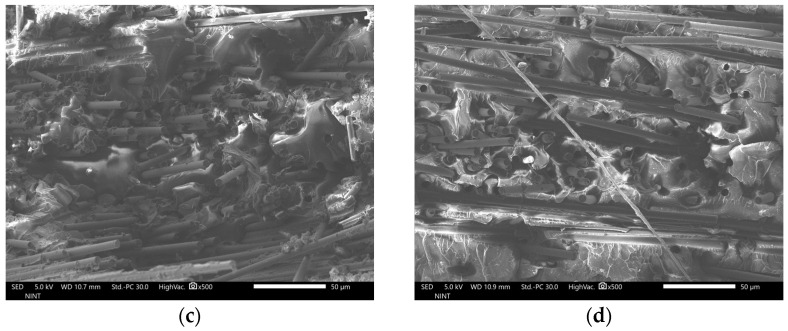
FE-SEM images of the rCFRP specimens, showing fractured surfaces, magnified 500 times, specifically for (**a**) 6 mm rCF-added composite specimen, (**b**) 12 mm rCF-added composite specimen, (**c**) 18 mm rCF-added composite specimen, and (**d**) 24 mm rCF-added composite specimen.

**Figure 4:**
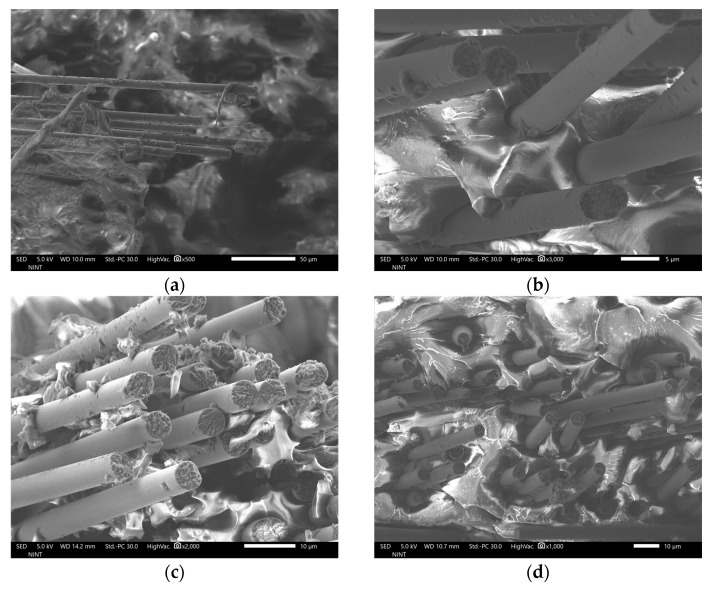
Magnified FE-SEM images of the fractured surface of rCFRP specimens: (**a**) 6 mm rCF-added composite specimen, (**b**) 12 mm rCF-added composite specimen, (**c**) 18 mm rCF-added composite specimen, and (**d**) 24 mm rCF-added composite specimen.

Figure 3a–d and Figure 4a–d show the FE-SEM images of the fractured surface of rCFRP specimens that have undergone tensile testing. While there are out-of-plane fibers, images show noticeable variation in fiber directions within the plane. Fibers appear well distributed within the matrix with a predominance of in-plane alignment. Regarding this, simplification by assuming the specimens are flat and fibers are in-plane oriented is reasonable. Dark holes in the matrix are evident in the images, which are likely voids or fiber pull-out sites. Debonding may yield to these holes, though further investigation could confirm the cause more in depth.

**Figure 5:**
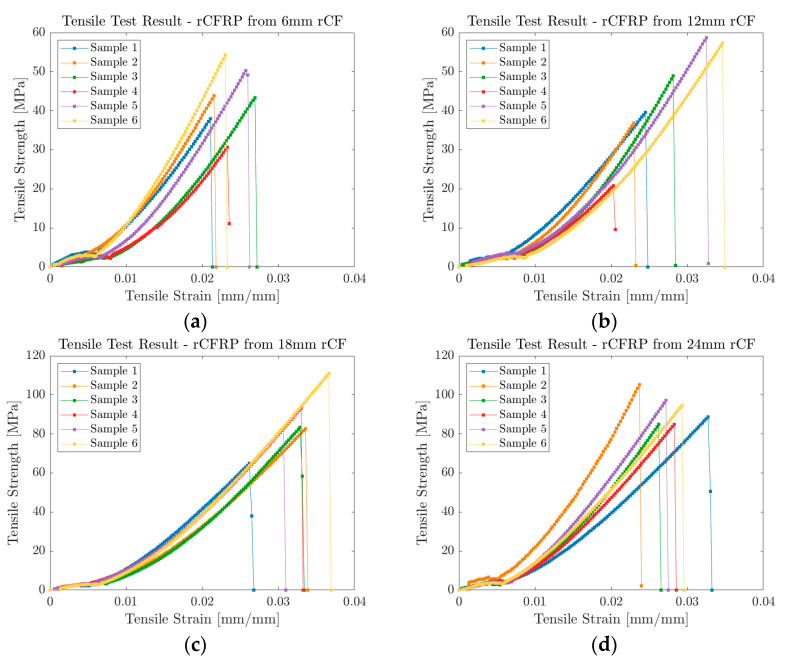
Stress–strain curves of the tested rCFRP laminate specimens: (**a**) 6 mm rCF-added composite specimen, (**b**) 12 mm rCF-added composite specimen, (**c**) 18 mm rCF-added composite specimen, and (**d**) 24 mm rCF-added composite specimen.

Figure 5a–d illustrates the recorded stress–strain curves of the tests. Samples from all the specimen groups exhibited good linear characteristics until tensile failure. Regardless of the fiber length, all specimens showed an immediate drop after reaching the ultimate tensile stress value. This sudden change in stress suggests brittle characteristics of the composites. While rCFRPs with 6 and 12 mm rCF reached its ultimate stress below 60 MPa, rCFRPs with 18 and 24 mm rCF reached their ultimate stress below 120 MPa.

**Figure 6:**
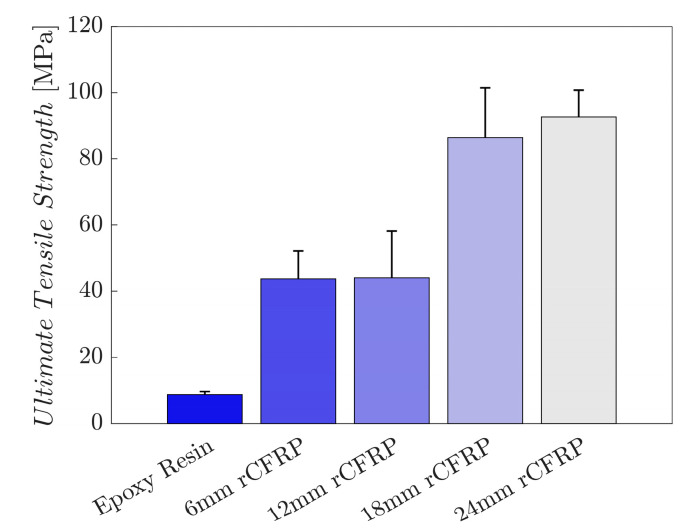
The relationship between rCF fiber length and ultimate tensile strength with error bars indicating one standard deviation above the mean.

The tensile strength values are shown in Figure 6. The results of six samples of each composite type were averaged. The bar graph displays the tensile strength, which increases along the length of the incorporated rCF; the longer the incorporated fiber length, the greater the ultimate tensile strength.

The mean tensile strength of the control specimens without rCF was 8.7 MPa. Specimens with 6 mm rCF showed a 401.2% increase to 43.7 MPa, while the values of those with 12 and 18 mm rCF reached 44.0 and 86.4 MPa, representing increases of 404.7% and 890.6%, respectively. Specimens with 24 mm rCF achieved 92.6 MPa, a 962.1% improvement over the control sample.

The diameter, length, volume fraction, and packing configuration of the fibers affected the mechanical properties of the fibrous composite materials. Fibers with high aspect ratios transfer stress more effectively than those with low aspect ratios, particularly in randomly oriented fibrous composites [91,92,93]. Fu and Lauke analytically confirmed the direct proportional relationship between the fiber length and tensile strength of short fiber-reinforced polymer composites [94]. Van der Werken confirmed the influence of the fiber length of rCFs on the tensile strength and modulus using an analytical approach [7,92]. Razaei et al. enhanced the thermal and damping properties of carbon-fiber-incorporated composites by increasing the fiber length [95].

Incorporating fibers with greater aspect ratios or lengths into composite materials significantly increases the tensile strength. This enhancement was primarily due to the increased effective fiber matrix and fiber–fiber contact area. The increase in fiber length allows more surface area for force transfer to both the polymer composite matrix and incorporated fiber reinforcement [96].

The increase in different fiber lengths, in other words, the higher fiber aspect ratio, provides a larger surface area for stress transfer between the fibers and the polymer matrix, enhancing the interfacial bonding and stress distribution. However, beyond a certain fiber length, issues such as fiber curling, bundling, and entanglement may impede stress transfer and overall mechanical performance. Therefore, balance in the fiber length for tensile strength optimization is supported by various studies, indicating that the optimal fiber length can vary depending on specific composite configurations and desired properties. The resultant tensile strength in certain systems has been reported; while 20 mm was determined to be the optimal fiber length to achieve maximum tensile strength, other studies reported that 6 and 7 mm are optimum [7,91,97,98].

**Figure 7:**
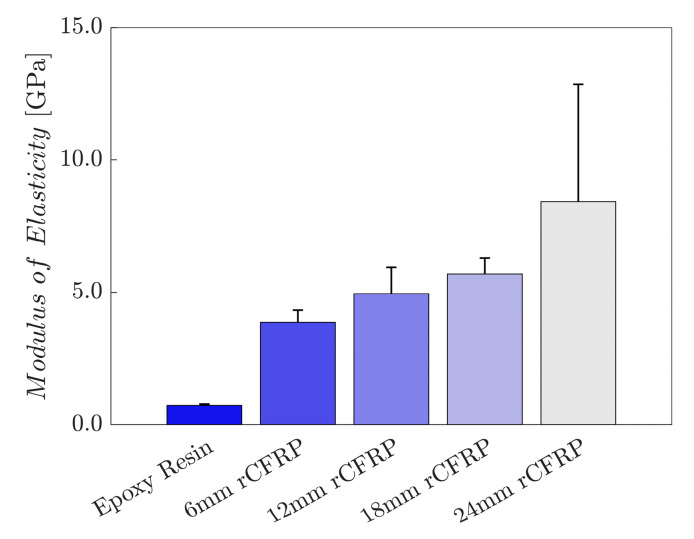
Modulus of elasticity as a function of the rCF fiber length examined via tensile tests.

Figure 7 shows the modulus of elasticity values of the rCF-incorporated epoxy composites; the values were calculated as the arithmetic means of six samples for each composite type. The bar graph presents a directly proportional relationship between the incorporated rCF length and the modulus of elasticity; the longer the incorporated fiber length, the greater the tensile modulus.

The average elastic modulus of the control specimens was 0.7 GPa. The 6 mm rCF-incorporated specimens exhibited a 432.4% increase, reaching 3.9 GPa. The values of specimens with 12 and 18 rCF reached 4.9 and 5.7 GPa, representing an increase of 581.6% and 685.5%, respectively. Furthermore, specimens with 24 mm rCF achieved 8.4 GPa, a 1061.1% improvement over the control.

The high variation in elastic modulus for 24 mm fiber composites likely results from fiber entanglement during fabrication. Longer fibers have larger aspect ratios, which correlates with higher caging numbers and greater entanglement. This leads to less uniform fiber distribution within the composite matrix, potentially causing variability in elastic modulus [99,100]. Nonetheless, the 24 mm specimen group’s mean value in Figure 7 well represents superior elastic modulus compared to other groups, suggesting it as the optimal fiber length for mechanical performance.

Similar to the tensile strength, the integrated fiber lengths also affected the tensile modulus results, with the same theoretical assumptions. Long fibers have greater aspect ratios and form dense fiber networks, leading to enhanced strength. However, beyond a certain length, long fibers can hinder stress transfer; thus, the elastic modulus of the composite decreases. These limitations in the ability of the fiber length to improve the mechanical performance of the composite have also been reported in the literature [7,91,92,94,97,101].

### 3.2. Piezoresistive Electrical Properties

#### 3.2.1. Initial Electrical Resistance

The initial electrical resistance provides insight into the electrical conductivity and internal structure of a composite in its natural state without an external load. Fibers in fibrous composites form an electrically conductive network and predominantly transmit electrical signals along the fiber direction, in most cases in the longitudinal direction [73,74,75].

Fiber length is key to understanding the mechanical and electrical properties of a composite [102]. Owing to their tendency to disperse randomly, short fibers create larger void volumes, which delays the electrons from traveling from fiber to fiber and makes the tunneling effect more challenging. Ultimately, this challenge increases the electrical resistance. In contrast, with their tendency to entangle or bundle, long fibers form dense networks with large contact areas, thus facilitating the movement of electrons. This relationship between the fiber length and the electrical resistance is inversely proportional [103].

The given constant weight fraction of the fiber reinforcements allows the initial electrical resistance to provide valuable information regarding the internal structure of the composite. Long fibers have more contact points per unit length, which allows long fibers to create continuous and efficient electrical pathways and therefore reduce resistance. Fibrous composites with randomly oriented and dispersed fiber networks have a high initial electrical resistance, whereas fiber-reinforced composites with aligned fibers have a comparatively low resistance [103].

Based on these principles, the electrical behavior of composites with varying fiber lengths can be hypothesized theoretically. The 6 mm rCF is anticipated to have the highest initial electrical resistance, with a progressive reduction in resistance as the fiber length increases to 12, 18, and 24 mm. The electrical properties of a composite are also related to its mechanical behavior. Composites with a high initial resistance may experience greater deformation, whereas low-resistance composites are expected to exhibit greater mechanical resistance.

**Figure 8:**
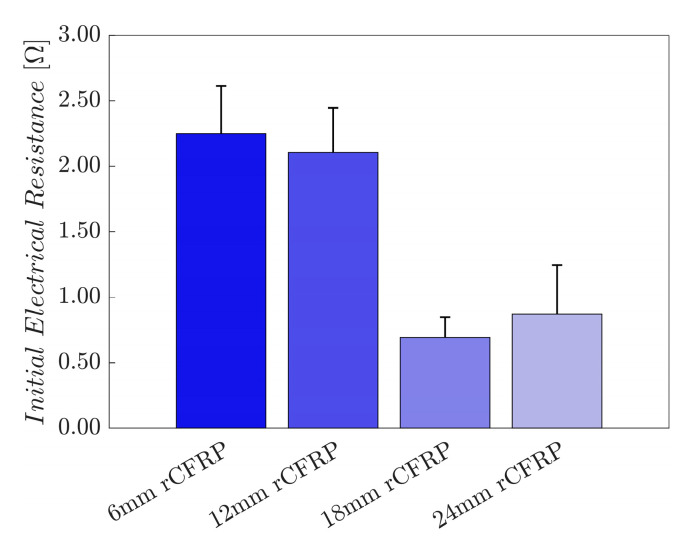
Initial electrical resistance as a function of the rCF fiber length.

Figure 8 shows the initial electrical resistance of the rCF-incorporated epoxy composites. These values were arithmetically averaged for four samples of each composite type. The resultant bar graph shows an inversely proportional relationship between the incorporated rCF length and the initial electrical resistance; the longer the incorporated fiber length, the smaller the resistance.

The average initial electrical resistance of the control specimens that did not incorporate rCF was excluded owing to their insulating characteristics. The 6 mm rCF-incorporated specimens exhibited a value of 2.2488 Ω, while those with 12 and 18 mm rCF reached 2.1070 and 0.6910 Ω, representing a decrease of 6.3% and 69.3%, respectively. Specimens comprising 24 mm rCF achieved 0.8715 Ω—a 61.2% decrease over the specimens with 6 mm rCF. The results agree with the hypothesis based on the theoretical relationship between fiber length and electrical resistance from the literature.

These findings have significant implications for composite designs, particularly in applications where electrical conductivity is a key consideration. As a result, balancing the fiber length with other desired properties is crucial for optimizing composite performance for self-sensing applications.

#### 3.2.2. Average Maximum Electrical Resistance Change Rate under Cyclic Loading Test

**Figure 9:**
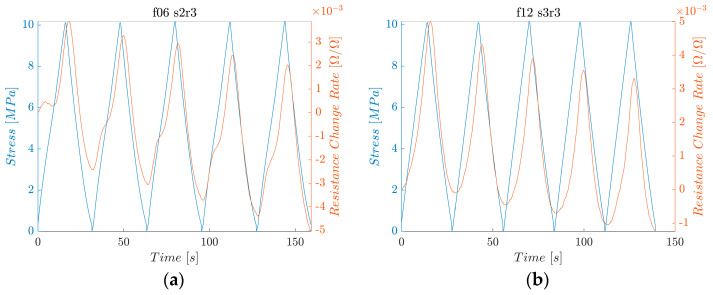

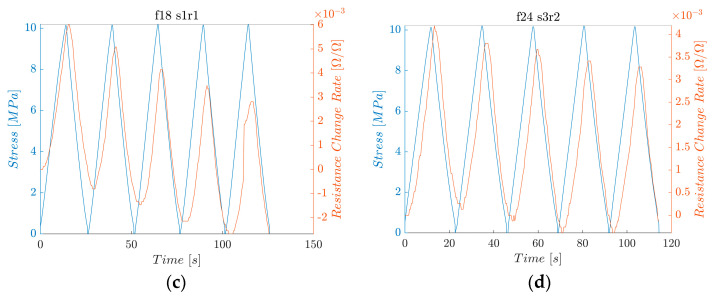
Electrical resistance and applied stress of the (**a**) second sample of rCFRP from 6 mm rCF; (**b**) third sample of rCFRP from 12 mm rCF; (**c**) first sample of rCFRP from 18 mm rCF; and (**d**) third sample of rCFRP from 24 mm rCF.

The strain sensitivity was quantitatively assessed using the average maximum electrical resistance change rate. In addition, the fractional change in the electrical resistance, depending on the literature, and the mean maximum electrical resistance change rate are calculated. Figure 9 shows the electrical resistance change rate (orange) and the applied stress (blue) as functions of time. The electrical resistance follows the pattern of the applied tensile stress: the resistance increases as the stress increases and decreases as the stress decreases. Similar phenomena have also been reported for carbon-nanomaterial-added CFRP composites [104,105].

**Figure 10:**
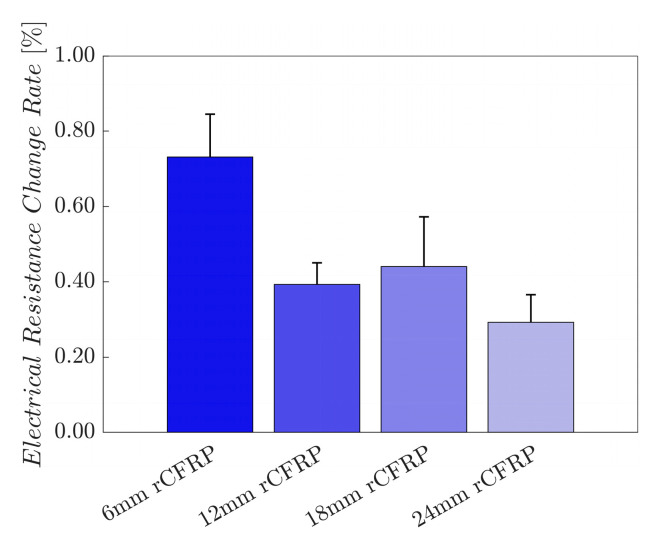
Maximum electrical resistance change rate as a function of the rCF fiber length.

Figure 10 shows the electrical resistance change rates of the rCF-incorporated epoxy composites. These values were arithmetically averaged for four samples of each composite type. The resultant bar graph shows an inversely proportional relationship between the incorporated rCF length, and the electrical resistance change rate; the longer the incorporated fiber length, the smaller the change rate.

The average maximum electrical resistance change rate of the 6 mm rCF-incorporated specimens was 0.7315%, while those of the 12 and 18 mm rCF-incorporated samples were 0.3930% and 0.4400%. Specimens with 24 mm rCF achieved 0.2923%. The 6 mm rCF specimens exhibited the highest mean maximum electrical resistance change rate, followed by the 18, 12, and 24 mm rCFRP specimen groups.

The average maximum electrical resistance change rate represents the amplitude of the change in electrical resistance under applied stress. This parameter signifies the electrical signal strength for each specimen group, as the uniaxial tensile load applied to all specimens was constant. A high value implies a greater change in resistance for a given constant external load, characterizing the sensitivity of the material to the external force.

Composites comprising shorter rCFs under tensile loading demonstrated a greater change in resistance than those with longer rCFs. This phenomenon can be attributed to the ease with which the electrically conductive network breaks within shorter fiber-incorporated composites. As the material deforms mechanically, the rCF reorients itself within the polymer matrix, altering the conductive network through mechanical strain. Short fibers are more likely to be pulled apart, increasing the fiber-to-fiber gap and, thus, the electrical resistance. Conversely, long fibers form stable conductive and stress-transmitting networks. When subjected to external forces, composites incorporating longer rCFs are less likely to deform mechanically owing to entanglement and bundling. While the fibers are pulled away longitudinally and brought closer transversely, the change in the conductive network is less pronounced compared with composites comprising shorter rCFs [104,106,107,108].

These observations supported the hypothesis that composites incorporating longer rCFs exhibited lower electrical resistance change rates than those incorporating shorter rCFs when subjected to tensile loading. This mechanism explains the trend observed in our experimental results, where longer rCF lengths correspond to lower maximum electrical resistance change rates.

#### 3.2.3. Gauge Factor

For a quantitative assessment of the strain sensitivity, the gauge factor was calculated using the electrical resistance change rate and strain data. The gauge factor is the rate of change in electrical resistance per unit strain. In particular, the gauge factor shows the relationship between the electrical and mechanical strains, which is calculated as follows:

(1) $$k=\frac{R_{f}}{\varepsilon }=(\frac{\Delta R}{R_{0}})/(\frac{\Delta L}{L_{0}})$$

where k is the gauge factor, $R_{f}$ is the electrical resistance change rate, $R_{0}$ is the initial electrical resistance, and ε is the strain. In comparison, a composite with a greater gauge factor reacts electrically to mechanical deformation with enhanced sensitivity.

The aspect ratio of the fibers is an important factor in network formation. Owing to their larger aspect ratios, longer fibers can form more effective conductive paths. A higher aspect ratio increases the chances of the fibers connecting with each other and creating a more extensive conductive network.

**Figure 11:**
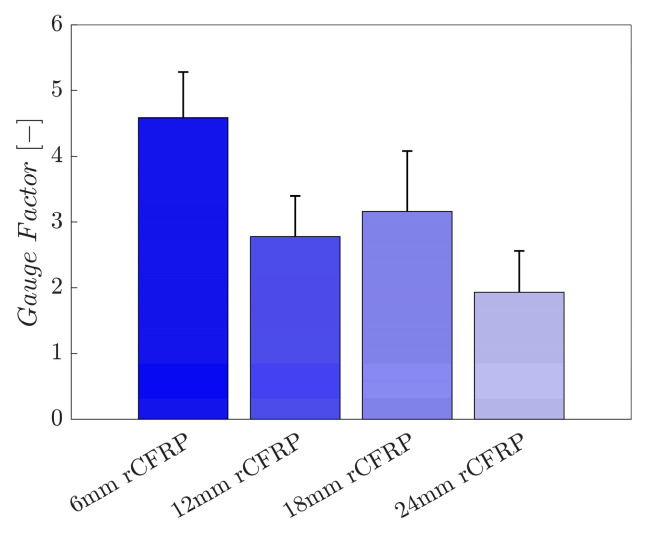
Gauge factor as a function of the rCF fiber length.

Figure 11 shows the gauge factors of the rCF-incorporated epoxy composites. These values were arithmetically averaged for four samples of each composite type. The resultant bar graph shows an inversely proportional relationship between the incorporated rCF length and strain sensitivity; the longer the incorporated fiber length, the smaller the gauge factor.

The average gauge factor of the 6 mm rCF-incorporated specimens was 4.5876, while those of the specimens with 12 and 18 mm rCF were 2.7786 and 3.1630, representing a decrease of 39.4% and 31.1%, respectively. Specimens with 24 mm rCF exhibited a value of 1.9306—a 57.9% deterioration over the specimens incorporated with 6 mm rCF. The 6 mm rCF specimens exhibited the highest mean gauge factor, followed by the 18, 12, and 24 mm rCFRP specimen groups.

The composites with shorter rCFs exhibited higher gauge factor values than those with longer fibrous fillers. This result is consistent with the mean maximum electrical resistance change rate, suggesting that shorter fibrous composites form less effective electrically conductive networks. The fiber-to-fiber gaps of these composites spread out under mechanical strain, which leads to an increase in the electrical resistance and change rates.

In contrast, longer fibers create smaller gaps between themselves and have a greater ability to attach to each other owing to their length. Consequently, longer fibers encounter minor mechanical strain and are less likely to separate from each other, which leads to the relatively higher modulus of elasticity shown in Figure 7. A small strain value leads to a smaller electrical resistance change rate and, consequently, a smaller gauge factor value.

#### 3.2.4. Coefficient of Determination

The coefficient of determination—also known as the R-squared value—was calculated through linear regression between the calculated stress and electrical resistance change rate to assess the sensing stability [104,106,108,109]. The degree of data scattering between the applied stress and the electrical resistance change rate in each specimen was determined by examining the R-squared values. When the discrepancies between the actual data and corresponding fitted data increased, the R-squared value decreased. An R-squared value near 1.0 displays less scattering of data and a strong regularity, whereas the R-squared value decreases as the data scattering increases.

**Figure 12:**
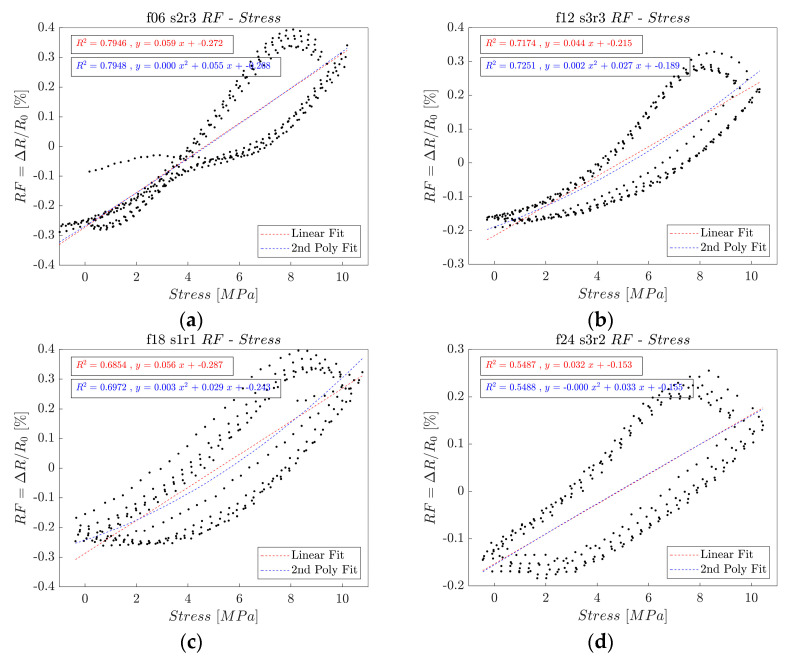
R-squared results of electrical resistance and applied stress for the (**a**) rCFRP from 6 mm rCF, (**b**) rCFRP from 12 mm rCF, (**c**) rCFRP from 18 mm rCF, and (**d**) rCFRP from 24 mm rCF.

Figure 12 shows the linear and polynomial regressions shown by the red and blue lines, respectively, for the electrical resistance change rate along the y-axis and the applied stress along the x-axis.

**Figure 13:**
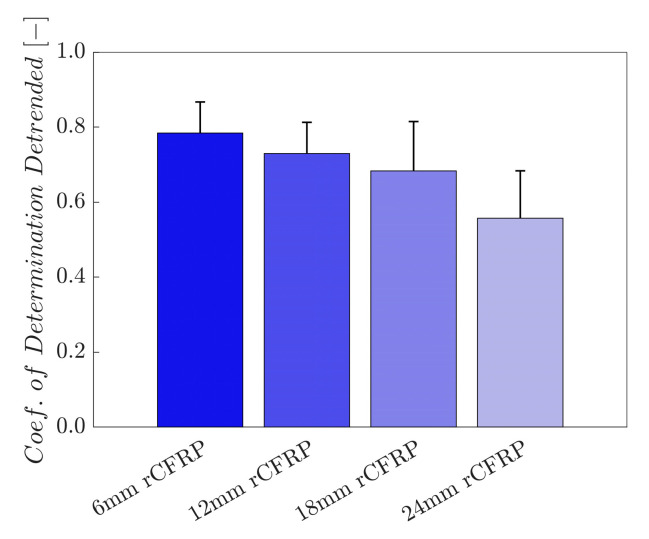
Coefficient of determination as a function of the rCF fiber length.

Figure 13 shows the coefficients of determination derived from the rCF-incorporated epoxy composites. The resultant bar graph presents an inversely proportional relationship between the incorporated rCF length and the coefficient of determination; the shorter the incorporated fiber length, the greater the R-squared value for the self-sensing composite.

The average R-squared value of the 6 mm rCF-incorporated specimens was 0.7841, while those of samples with 12 and 18 mm rCF reached 0.7295 and 0.6830, respectively. Specimens incorporating 24 mm rCF reached a value of 0.5563. The 6 mm rCF specimens exhibited the highest mean R-squared value, followed by the 12, 18, and 24 mm rCFRP specimen groups. Notably, the composites incorporating 6 mm rCF exhibited the best piezoresistive sensing characteristics in terms of the R-squared value and gauge factor.

#### 3.2.5. Peak Shift

Peak shift analysis was performed to compare the sensitivities of the fabricated fiber-reinforced composites in the time domain. Peak shift was calculated using the following equation:

(2) $$\mathrm{peak}\;\mathrm{shift}\left(\%\right)=\frac{\Delta t}{t_{p}}\times 100$$

where Δt is the delayed time interval between the applied stress peak and the electrical resistance peak, and $t_{p}$ is the elapsed time from the initial point of electrical resistance change rate cycle to the peak point [104,106,107,108,109]. High values of peak shift show a slow response in the conversion from mechanical deformation to electrical signals, whereas low values indicate electrical signals according to the change in the applied stress [104,106,107,108,109].

**Figure 14:**
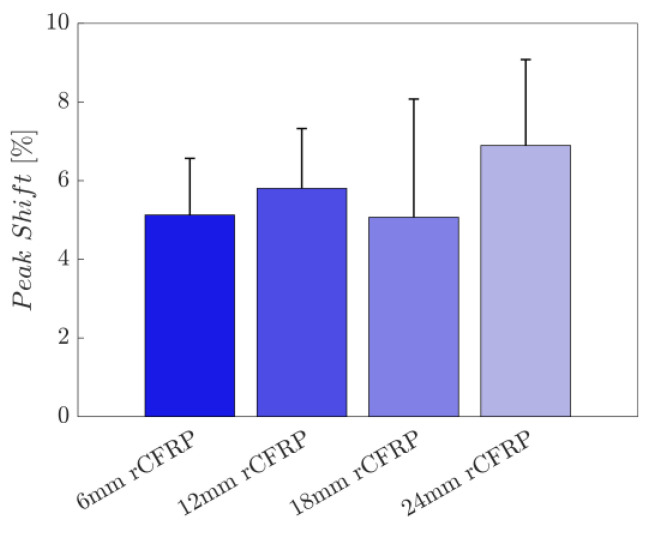
Peak shift as a function of the rCF fiber length.

Figure 14 shows the peak shift values derived for the rCF-incorporated epoxy composites. These values were arithmetically averaged for four samples of each composite type. The resultant bar graph presents the direct proportional relationship between the incorporated rCF length and the time-domain sensitivity; the longer the incorporated fiber length, the longer the time delay for the self-sensing composite.

The average peak shift of the 6 mm rCF-incorporated specimens was 5.1268, and those of the with 12 and 18 mm rCF were 5.8028 and 5.0685, respectively; specimens with 24 mm rCF showed a value of 6.8941. The 6 mm rCF specimens exhibited the lowest mean peak shift, followed by the 18, 12, and 24 mm rCFRP specimen groups.

The small peak shift indicated that the resistance curve changed promptly in accordance with the change in the applied stress. Figure 14 shows the peak shifts of the rCF-incorporated epoxy composites. Each value was calculated by averaging the peak shifts of the four replicated samples for each type of composite. Recalling the sensing stability (R-squared) results (Figure 13), the composite types exhibiting high sensing stability exhibited a low peak shift, indicating good time-based sensitivity. The high matching relationship between the electrical resistance change and the applied stress, which was shown by the high R-squared value, was closely related to the time-based sensitivity.

#### 3.2.6. Averaged Maximum Electrical Resistance Change Rate under Low Fatigue Test

**Figure 15:**
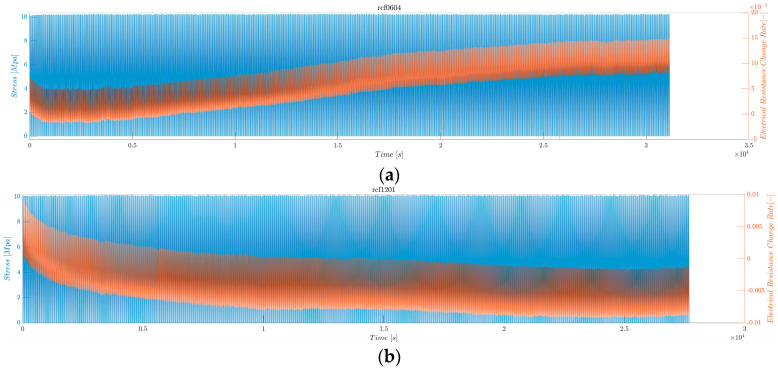

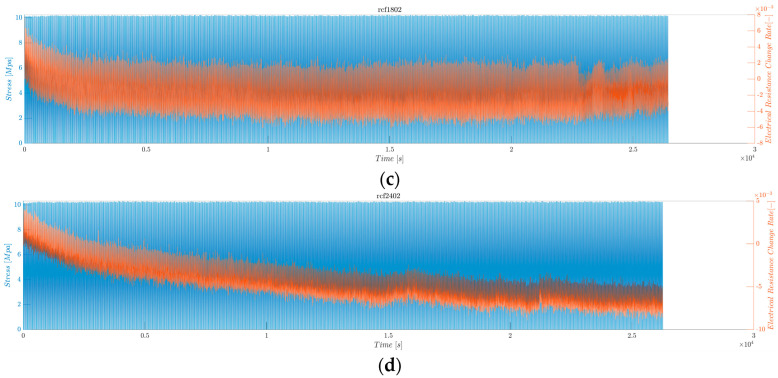
Electrical resistance change rate and applied stress under low fatigue test. (**a**) rCFRP from 6 mm rCF, (**b**) rCFRP from 12 mm rCF, (**c**) rCFRP from 18 mm rCF, and (**d**) rCFRP from 24 mm rCF.

The continuous sensing characteristics of the rCF-incorporated composites were verified through electrical resistance measurements under a low-fatigue tensile test, in which 1000 cycles of a maximum tensile loading of 10 MPa were applied. Figure 15 shows the resulting stress and electrical resistance change histories of the rCF-incorporated composites. Samples of all lengths demonstrated consistent and stable increases and decreases in electrical responses to the respective changes in tensile loading and unloading.

**Figure 16:**
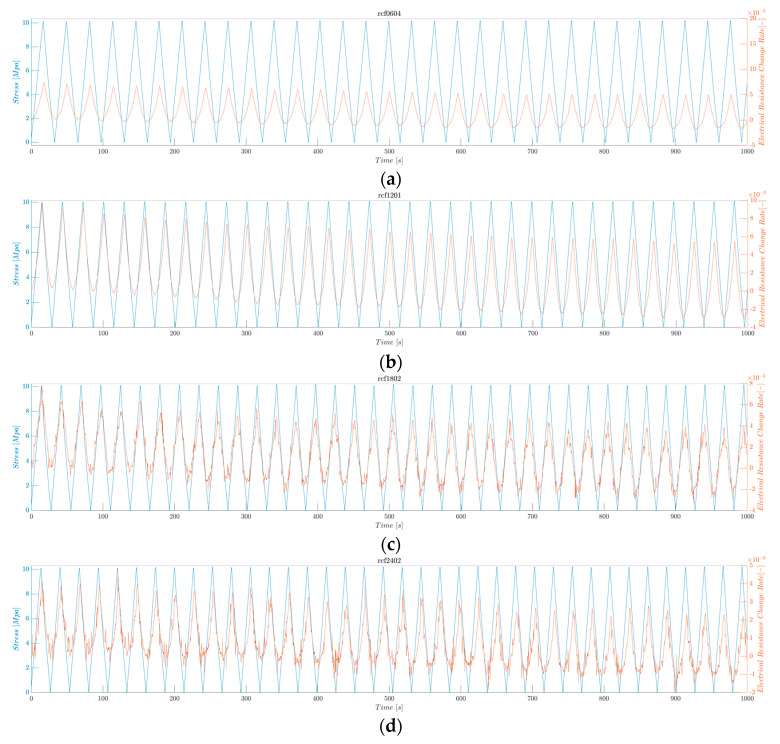
Electrical resistance change rate and applied stress under low fatigue test, showing the first 1000 s. (**a**) rCFRP from 6 mm rCF, (**b**) rCFRP from 12 mm rCF, (**c**) rCFRP from 18 mm rCF, and (**d**) rCFRP from 24 mm rCF.

**Figure 17:**
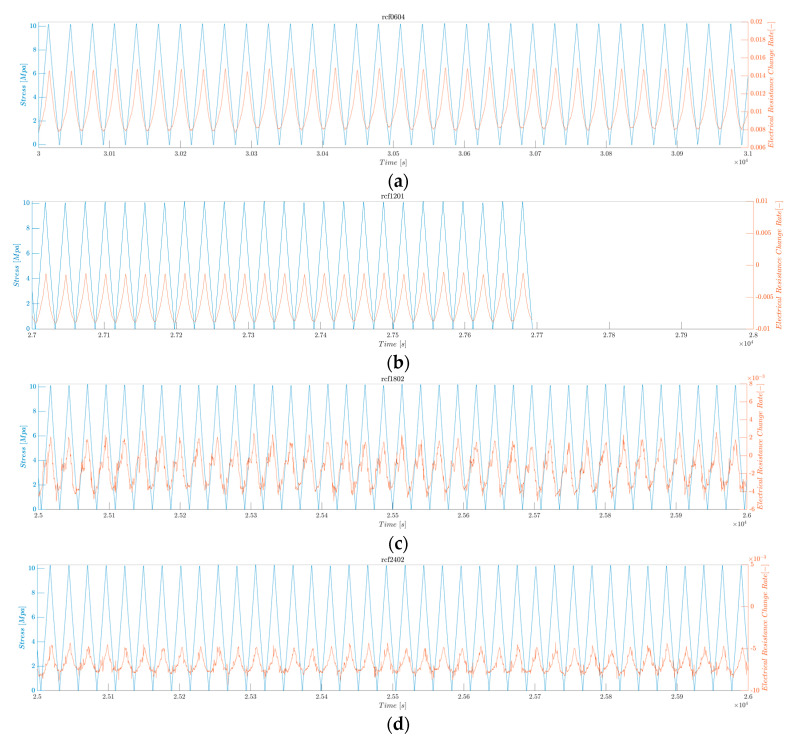
Electrical resistance change rate and applied stress under low fatigue test, showing the last 1000 s. (**a**) rCFRP from 6 mm rCF, (**b**) rCFRP from 12 mm rCF, (**c**) rCFRP from 18 mm rCF, and (**d**) rCFRP from 24 mm rCF.

Figure 15 shows the electrical resistance change rate (orange) and applied stress (blue) as functions of time. The electrical resistance follows the pattern of the applied tensile stress: the resistance increases as the stress increases and decreases as the stress decreases. This result can be confirmed in detail in Figure 16 and Figure 17, where the first 1000 s and last 1000 s of the test are plotted.

**Figure 18:**
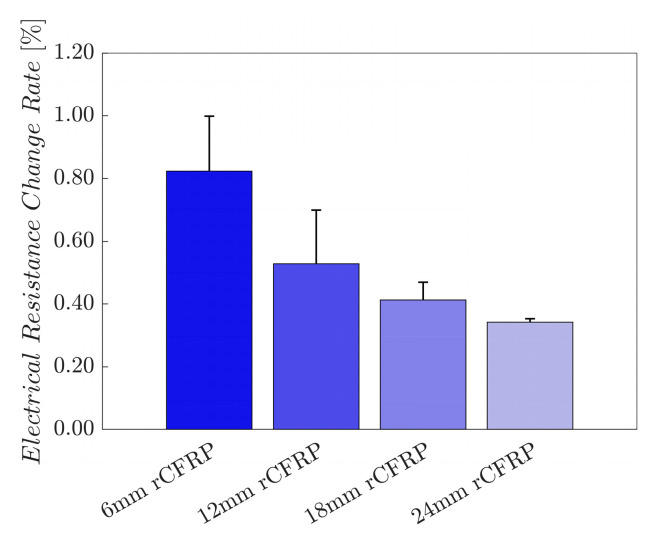
Maximum electrical resistance change rate in electrical resistance under low fatigue test as a function of the rCF fiber length.

Figure 18 shows the electrical resistance change rates of the rCF-incorporated epoxy composites during the low fatigue test. These values were arithmetically averaged for four samples of each composite type. The resultant bar graph shows an inversely proportional relationship between the incorporated rCF length and the electrical resistance change rate; the longer the incorporated fiber length, the smaller the change rate. This finding is consistent with the five cyclic loading test results, implying consistency regardless of the number of cyclic loads.

All samples demonstrated a steady decline in resistance during the low fatigue test, after which they exhibited steady increases and decreases in resistance. This phenomenon can be attributed to the relaxation of the rCF during the initial cycling, resulting in a higher contact between the rCF and, ultimately, a lower initial resistance [108]. The initial electrical resistance of each cycle increased owing to the accumulation of mechanical strain in the microstructures, following a decreasing logarithmic trend, which is consistent with the literature reviews of other composites under fatigue loading. Modeling and computational simulations of such trends are beyond the scope of this research; thus, it is a subject for further study.

According to Mu et al. and Zhang et al., changes in the polymer chains and conductive networks are responsible for the observable drift or decay of piezoresistive composite materials [110,111]. The conductive network appeared to play a greater role than the matrix viscoelasticity in the electrical resistance response. According to Boland et al., this behavior can be interpreted as the rapid deformation of the network, breaking the filler–filler connections, thus increasing the resistance [112]. The mobility of the fillers allows the network to relax slowly, reform connections, and decrease the resistance, which can be considered a self-healing process.

## 4. Concluding Remarks

This study investigated the influence of rCF fiber length on the mechanical and electrical properties of rCFRP composites. The findings revealed a significant trade-off between mechanical reinforcement and electrical sensing capabilities as a function of fiber length. The following are the concluding remarks based on the experimental results:

1. rCF incorporation significantly improved the mechanical properties of the EP composites. The 24 mm rCFRP showed greater tensile strength and modulus of elasticity, whereas the 6 mm rCFRP showed inferior results. The fiber length and mechanical properties exhibited a positive proportional relationship.
2. Regardless of the fiber length, the incorporated rCFs exhibited piezoresistive behavior. The conductive filler, rCF, forms a conductive network in the insulating EP composites and enables sensing capabilities. Therefore, the rCFRP composites have the potential to be used as self-sensing structural materials.
3. While the 24 mm rCFRP exhibited better electrical conductivity, the 6 mm rCFRP outperformed the piezoresistive performance with higher sensitivity (gauge factor), larger response magnitude (electrical resistance change rate), better linearity (R-squared values), and consistent time-domain sensitivity (peak shift). The fiber length has an inverse relationship with the initial electrical resistance and piezoresistive sensing properties, including the electrical resistance change rate, gauge factor, peak shift, and R-squared value.
4. All the composites exhibited stable and consistent electrical responses under cyclic and fatigue loading. The reliability of the rCF-incorporated composites for long-term sensing applications was demonstrated.

The results of this study can contribute to the establishment of guidelines for manufacturing highly stable and sensitive piezoresistive rCF-incorporated epoxy resin composites. Simultaneously, it establishes the potential of rCF in composites with both structural and sensing capabilities. Therefore, this study contributes to the development of sustainable and multifunctional materials, and the findings support the circular economy concept by showcasing high-value applications for recycled carbon fibers, potentially driving increased recycling efforts in the composite industry.

However, the effects of various environmental conditions on the piezoresistivity of rCFRP composites have not yet been investigated. The percolation threshold was not examined as a function of fiber length and content. Moreover, investigations of the long-term performance of rCF/EP composites have not been reported to date. Therefore, from the standpoint of the practical applications of composites, further studies will be carried out along these lines.

## Figures and Tables

**Table 1:** Physical properties of epoxy resin.

Property	Epoxy Resin	Unit
Tensile Strength	72	MPa
Tensile Modulus	3170	MPa
Flexural Strength	125	MPa
Compressive Strength	97	MPa
Compressive Modulus	2377	MPa
HDT	62	°C
Viscosity	450	mPas @ 25 °C
Specific Gravity	1.11	g/mL @ 25 °C

**Table 2:** Physical properties of recycled carbon fiber.

Property	Recycled Carbon Fiber	Unit
Fiber Diameter	5.2–6.8	µm
Fiber Density	1.5	g/cm^3^
Tensile Strength	<3500	MPa
Tensile Modulus	<240	GPa
Elongation	<1.61	%
Carbon Content	<95	wt.%
Electrical Resistivity	1.23 × 10^−3^	Ω⋅cm

**Table 3:** Average thickness variation of the specimens.

Sample Group	Average Thickness [mm]	Standard Deviation
6 mm	2.585	±0.261
12 mm	2.567	±0.240
18 mm	2.371	±0.289
24 mm	1.874	±0.493

## Data Availability

The data presented in this study are available on request from the corresponding author. The data are not publicly available to prevent indiscreet replications.
